# Terahertz Portable Handheld Spectral Reflection (PHASR) Scanner

**DOI:** 10.1109/access.2020.3045460

**Published:** 2020-12-17

**Authors:** ZACHERY B. HARRIS, MAHMOUD E. KHANI, M. HASSAN ARBAB

**Affiliations:** Department of Biomedical Engineering, Stony Brook University, Stony Brook, NY 11794, USA

**Keywords:** Terahertz imaging, terahertz time-domain spectroscopy

## Abstract

We report on the development and characterization of a handheld terahertz (THz) time-domain spectroscopic scanner for broadband imaging between approximately 0.25 and 1.25 THz. We designed and fabricated a 3D-printed fiber-coupled housing which provides an alignment-free strategy for the placement and operation of the THz optics. Image formation is achieved through telecentric beam steering over a planar surface through a custom f-*θ* scanning lens. This design achieves a consistent resolution over the full 12 × 19 mm field of view. Broadband spectral imaging is demonstrated using a 1951 United States Air Force Resolution Test Target. The consistency of the resolution over the wide field is validated through Boehler Star resolution measurements. Finally, a practical scenario of subsurface imaging on a damaged section of an aircraft wing is demonstrated. The THz PHASR is a field-deployable imaging system with the versatility to be applied to a much broader range of targets and imaging scenarios than previously possible, from industrial non-destructive testing to clinical diagnostic imaging.

## I. INTRODUCTION

Technologies using terahertz (THz) frequency light have been adapted to a broad range of applications from security screening [1] and non-destructive testing [2]-[4] to biomedical analysis [5]-[7]. Accordingly, a wide variety of imaging modalities have been developed to serve these purposes. However, portable THz systems are still in their early stages of development. Many investigations use single-pixel techniques such as raster scanning the target in front of a stationary THz time-domain spectroscopy (THz-TDS) setup to form spectral images [8]-[11]. This strategy is limited by problems of alignment and phase ambiguity in reflective imaging and is only feasible for small, easily movable samples [12]. Existing THz cameras rely on bolometric measurement [13]-[16] or use active [17] or passive [18] THz power detection, neither of which provide spectroscopic information. Meanwhile, efforts towards spectroscopic array detectors [19], [20] and grating-based camera systems [21] only provide single-axis line scans and require linear translation stages to produce two-dimensional images. Single-pixel, compressive sensing techniques can acquire spectral images but require the full target area to be contained within the collimated beam [22]-[24]. Systems in which a single-detector imaging head is raster scanned over a stationary target [5], [25], [26] suffer from alignment and size limitations similar to moving-target systems, but have paved the way for the development of more portable THz devices [12], [27]-[29]. A single-point THz spectroscopy system, called Micro-Z, has been demonstrated in a battery-powered handheld form factor [27]. A larger prototype, called Mini-Z, has been used for dielectric measurements in biomedical samples [29] and sub-surface imaging in art preservation applications [12]. Also, inexpensive quasi time-domain spectroscopy systems have been demonstrated using THz sources pumped by laser diodes at 1550 nm wavelength, used commonly in telecom applications, [30], [31] for field-deployable battery-powered outdoor measurements [32]. Other realizations of a handheld scanning device use a pivoting mirror to steer the THz beam across a sample, providing 1D line scans [33], [34]. An alternative approach to beam steering was demonstrated in benchtop setups using a scanning lens and two separate mirrors for each orthogonal scan direction to provide fast, time-resolved, 2D terahertz imaging [3], [35]. Finally, the combination of a polygonal mirror and an f-*θ* focusing lens have been used for sub-THz beam steering and transmission imaging [4]. However, these configurations did not form a telecentric imaging system, leading to scanning inaccuracy.

Here, we present the THz PHASR scanner: a Portable HAndheld Spectral Reflection scanner, which can record 2D THz-TDS images at 100 traces/s without phase ambiguity using a mirror mounted in a 2-axis motorized gimbal and a custom f-*θ* focusing lens. We describe the “alignment-free” design and show consistent resolution over the device’s field of view (FOV) before demonstrating its use for non-destructive testing by examining sub-surface features of an aerospace sample. We envision applications of such a handheld scanner in many previously prohibitive THz imaging scenarios, especially in cases such as *in vivo* biomedical imaging and non-destructive testing in production lines, where field-deployable devices are needed.

## II. DESIGN

**FIGURE 1.**
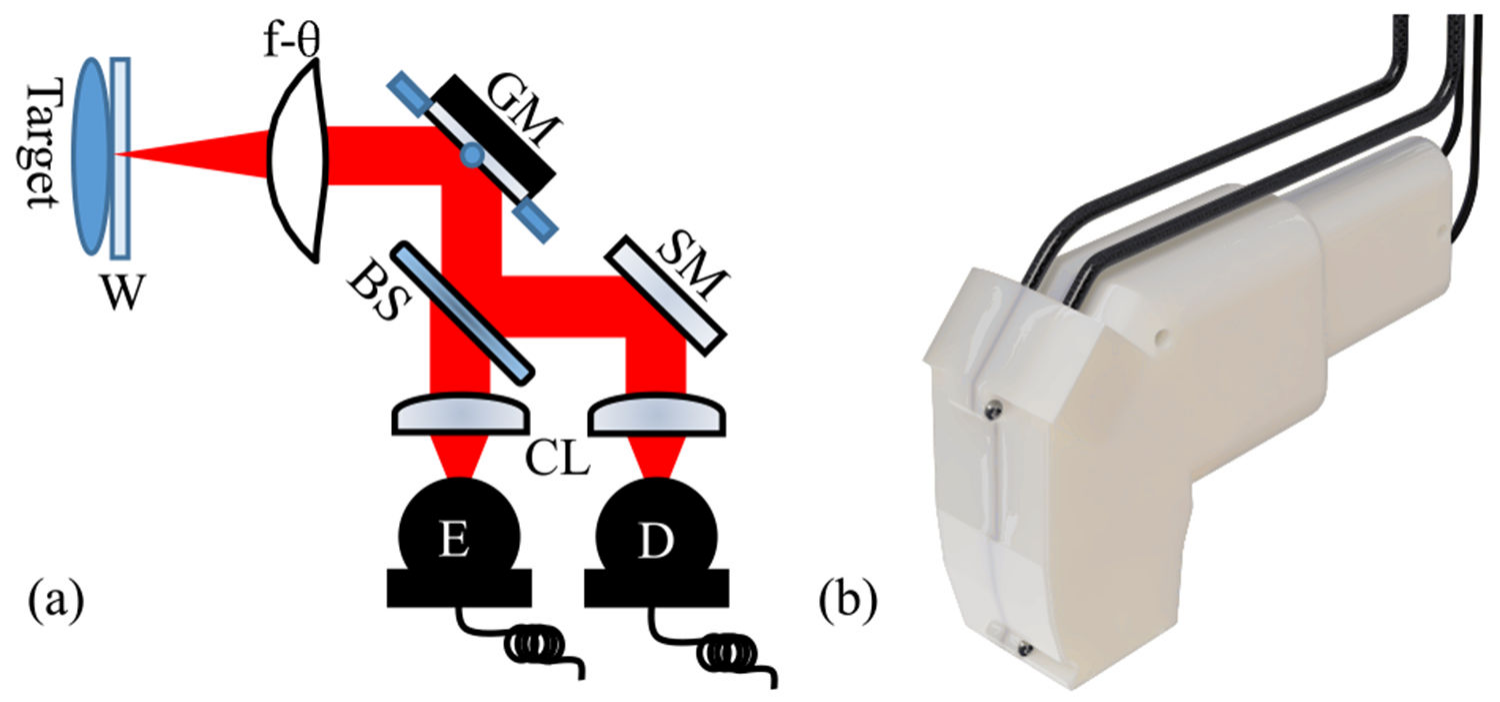
The THz PHASR scanner. (a) Diagram of optical setup. GM, gimballed mirror; W, imaging window; BS, beam splitter; SM, stationary mirror; CL, focusing and collimating lenses; D, detector PCA; E, emitter PCA. (b) Rendered image of the scanner.

A schematic of the THz PHASR scanner is shown in Fig. 1. Briefly, the THz beam is generated and detected by photoconductive antennas (PCA). The collimated beam passes through a beam splitter and is steered using a mirror mounted on a motorized gimbal (T-OMG, Zaber Technologies Inc. Vancouver, BC, Canada). The mirror gimbal is located such that the mirror rotates about the rear focus of a custom-made, 40 mm focal length, high-density polyethylene f-*θ* lens, which focuses the beam on the target with normal incidence. This telecentric arrangement also has the property that the focused beam maintains a constant spot-size over the FOV [36]. Finally, the reflected beam is directed towards the detector by the beam splitter. Telecentric beam steering is ideal for a handheld scanner because the scanning mechanism consists of a single mirror mounted in a commercially available gimbal, which is small in size and does not require complicated meta-surface design considerations. This arrangement thus provides accurate 2D scanning, which can be integrated into a self-contained device.

In order to keep the design compact and user-friendly, a stationary mirror was added to the detector path so that it sits parallel to the emitter, forming a single convenient handle for the device. To improve the design’s performance for practical samples, which may be malleable or liquid, an optional imaging window can be placed at the focus of the device in order to maintain a flat target surface. This window can also be used for self-calibration, background signal removal, and improved material parameter estimation [37]. The emitter and detector are part of a commercial asynchronous optical sampling system (TERA ASOPS, Menlo Systems Inc., Newton, NJ, USA), which controls data acquisition. A cable bundle carrying the 1550 nm fiber-optic cables along with the power and data cables for the motor and photoconductive antennas (Menlo Systems Inc.) connects the device to the computer and laser systems.

**FIGURE 2.**
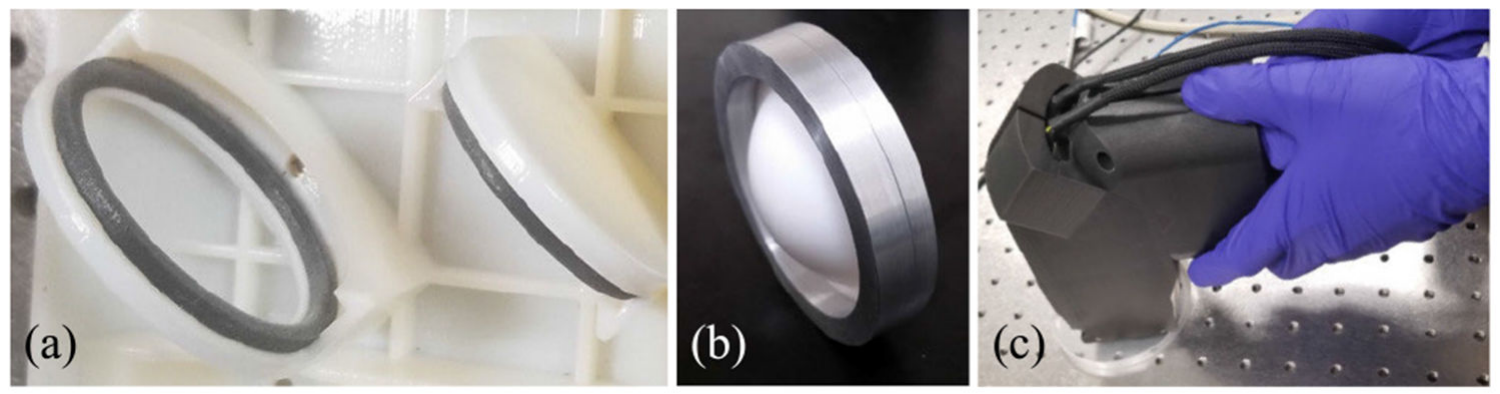
(a) Integrated mounts for beam splitter (left) and stationary mirror (right) with flexible printed padding (gray color). (b) Lens contained in custom metal collar for accurate z-axis positioning. (c) Photo of scanner in use.

The housing was 3D printed in a photopolymer plastic with a positional tolerance of ±0.001 mm/mm. The design consists of two halves which enclose the free-space THz path. It was designed specifically to locate the optical elements shown in Fig. 1(a) such that it can achieve “alignment-free” operation. That is, the orientation and location of the parts are predetermined and maintained by the housing. For the unmounted optical elements, such as the silicon beam splitter, a flexible plastic material was printed as padding to create an *in situ* mount inside the housing. This padding, shown in Fig. 2(a), allowed the parts to be slid into place but held them in alignment as the scanner was used. All optics are mounted within the same half of the housing, with the other serving only as a lid to enclose them.

A custom metal collar, shown in Fig. 2(b), was fitted to the f-*θ* lens which correctly positioned the lens in the housing. Changes in lens design which preserve focal length then only require replacement of the collar instead of the entire housing. The handheld scanner, shown in use in Fig. 2(c), is 262 × 155 × 74 mm and about 1.1 kg total, with the housing itself contributing only approximately 510 g.

## III. PERFORMANCE EVALUATION

### A. DATA ACQUISITION AND PROCESSING

To acquire an image, the gimbaled mirror is steered such that the focused beam scans the image in a raster pattern using a distortion compensation method described previously [38]. The femtosecond lasers of the ASOPS system have a base repetition rate of 100 MHz and were set to a a difference frequency of 100 Hz giving an effective signal sampling rate of of 100 THz and recording speed of 100 waveforms/s. For each pixel, the average of 20 waveforms was recorded. Including beam steering overhead, scan time for each pixel is less than 0.3 seconds. The full FOV of this scanner is 12 × 19 mm with variable pixel size depending on the application and frequencies used for image reconstruction (typically around 1 mm). Both scan speed and FOV are limited by the commercial hardware used. As such this design provides a template for future instruments with further customized elements to improve scanning performance.

**FIGURE 3.**
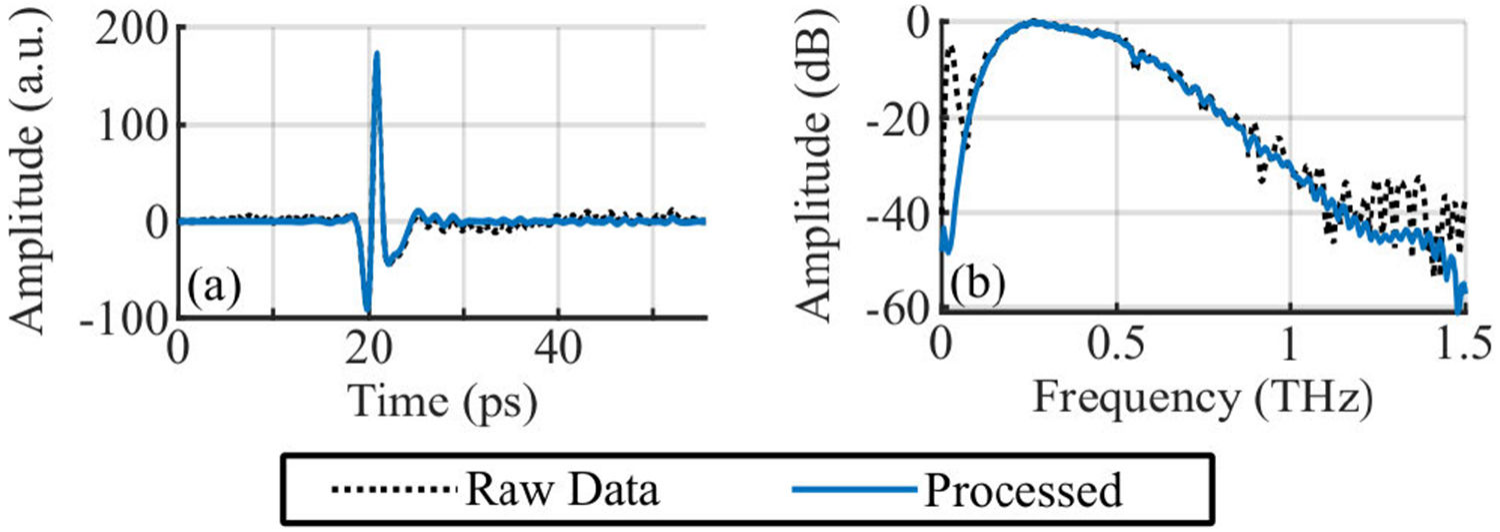
Examples of a reference signal (20 averages) before and after post-processing steps in (a) the time domain and (b) the frequency domain.

The post-processing steps consisted of applying a Gaussian high-pass filter (*μ* = 0 THz, *σ* = 0.05 THz) in the frequency domain followed by denoising using wavelet shrinkage with global thresholding. An example signal taken from a mirror can be seen in Fig. 3. The processed signal has a noise floor at about 1.25 THz at −45 dB. Where multiple reflections were present, a uniform set of manually defined windows were applied across all pixels to separate them. Spectral images are calculated from discrete Fourier transforms of the windowed signals.

### B. 1951 USAF RESOLUTION TEST TARGET

**FIGURE 4.**
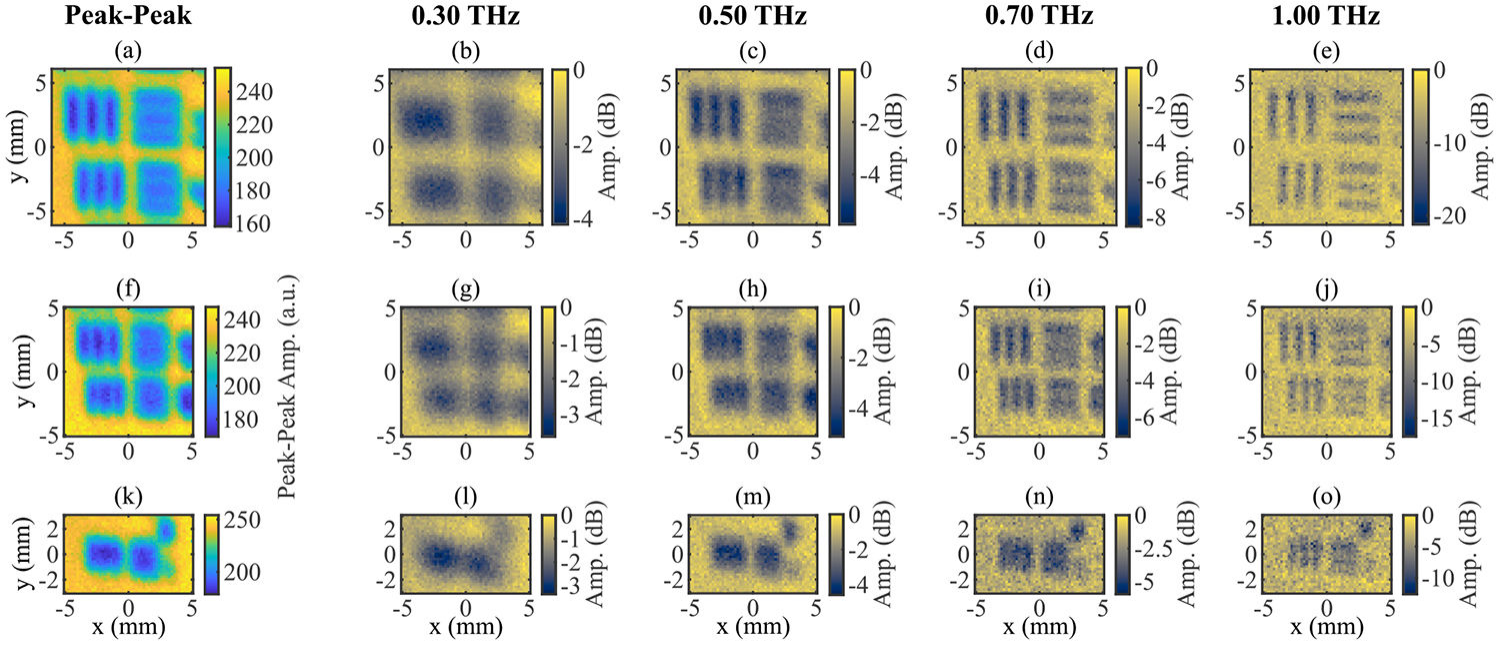
Images of 1951 USAF Resolution Test Target. (a-e) Group −1, Elements 3 and 4. (f-j) Group −1, Elements 5 and 6. (k-o) Group 0, Element 1. For each Element, images obtained using the Peak-Peak THz amplitude and selected frequencies are shown.

We assessed the resolution of the THz PHASR scanner using a standard chrome-on-glass United States Air Force (USAF) 1951 negative resolution test target. The line width of the smallest pattern for which the three parallel lines can be resolved provides an estimated resolution. Horizontal and vertical resolutions can be assessed separately using these images. Terahertz time-domain and spectral scans of the USAF resolution target are shown in Fig. 4. Because the cross-section of the emitted THz beam was non-circular, and thus the focus at the target was elliptical, there is a clear difference in the spatial resolution of the horizontal and vertical elements. For instance, in the peak-peak amplitude images, the smallest resolved feature is 0.56 mm in the horizontal direction and 0.71 mm in the vertical direction. The directional difference in the achievable spatial resolution persists at all frequencies, shown in 0.3, 0.5, 0.7, and 1.0 THz images in Fig. 4. This resolution is comparable or better than recent benchtop 2D spectral reflection imaging designs [24], [38]-[40].

The imaging resolution can be improved further by using appropriate post-processing techniques. For instance, we used the Wiener deconvolution technique to remove the blur in the spectral images caused by the non-zero focal diameter [41]. By modeling the 2D raster scan of the beam over the sample as a 2D convolution of object *o* with the beam point spread function (PSF) *h*, the image formed at each frequency is given by,

(1) i(x,y,f)=h(x,y,f)∗o(x,y)+n(x,y,f),

where *i* represents the image at frequency *f*, and *n* is noise corrupting the image. Here, Eq. (1) assumes a linear space-invariant imaging system was used, and *n* is represented by a zero-mean additive white Gaussian. Recovering the object function *o* from the blurred and noisy images *i* requires knowledge of the PSF *h*. Here, the PSF of the imaging system was modeled using the Gaussian beam distribution which depends on the frequency, the numerical aperture (NA) of the focusing lens, and the position of the target along the propagation axis [42]. However, a simple deconvolution resultant from dividing the Fourier transform of *i*(*x*, *y*, *f*) by the Fourier transform of *h*(*x*, *y*, *f*), i.e. *H*(*k_x_*, *k_y_*, *f*), will not result in satisfactory images when the noise variance is large and *H* is ill-conditioned [43]. To address this problem, using the Wiener deconvolution which minimizes the mean squared error between *i* and *o*, the Fourier transform of the object function can be estimated by [44],

(2) $$\hat{O}(k_{x},k_{y})=I(k_{x},k_{y},f)\;\;\;\times \frac{H^{*}(k_{x},k_{y},f)}{|H(k_{x},k_{y},f){|}^{2}+N(k_{x},k_{y})∕S(k_{x},k_{y})},$$

where H* is the complex conjugate of *H*, *I*(*k_x_*, *k_y_*, *f*) is the Fourier transform if *i*(*x*, *y*, *f*), *k_x_* and *k_y_* are the spatial frequency in the x and y directions, and *S*(*k_x_*, *k_y_*) and *N*(*k_x_*, *k_y_*) are the power spectral density of *o*(*x*, *y*) and *n*(*x*, *y*), respectively. Equation (2) requires exact knowledge of the spectral content of *o*(*x*, *y*) and *n*(*x*, *y*), which is not available. Here we estimate those values using the wavelet denoising algorithm, based on stationary wavelet transform of *i*(*x*, *y*, *f*) [45], [46]. In this approach, the median absolute deviation of the first-level (fine-scale) wavelet coefficients was used to estimate the noise power, and the variance of the denoised image as the object function’s mean power spectral density [47]. A flowchart describing the signal processing and image enhancement is given in the supplementary material.

**FIGURE 5.**
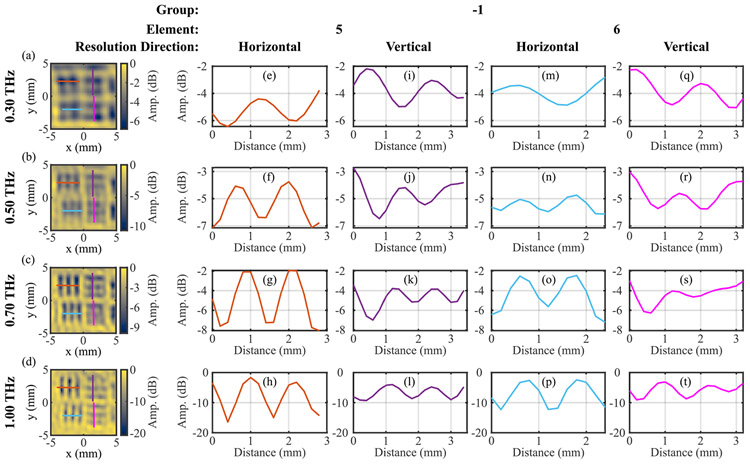
(a-d) Spatially deconvolved images at selected frequencies of Group −1, Elements 5 and 6 corresponding to Fig. 4(g-j). (e-t) THz amplitudes along the horizontal and vertical colored lines shown in (a-d) are plotted to demonstrate contrast in each image. The rows show results for 0.3, 0.5, 0.7 and 1 THz, respectively.

The results of the PSF modeling and image reconstruction are demonstrated in Fig. 5(a-d) showing the improved resolution in measurements from the USAF target group −1, elements 5 and 6, corresponding to Fig. 4(g-j). To further quantify the resolution, a line is drawn across each tribar pattern and the contrast of the pattern is calculated using,

(3) $$\text{Contrast}\;\%=\frac{|A_{\text{Max}}{|}^{2}-|A_{\text{Min}}{|}^{2}}{|A_{\text{Max}}{|}^{2}+|A_{\text{Min}}{|}^{2}},$$

where *A*_Max_ and *A*_Min_ are the maximum and minimum amplitudes within the path, respectively. Since the resolution pattern is composed of contrasting lines, the contrast ratio provides an easy to interpret and direct measurement of the performance. As an example, Fig. 5(e-t) show the amplitudes corresponding to the paths marked on Fig. 5(a-d). Tables 1 and 2 show the contrast along the same paths for the original images and after the PSF deconvolution reconstruction, respectively. Contrast for unresolved patterns are not quantified, including in cases of spurious resolution indicated by an inverted pattern with one less bar than expected such as in Fig. 5(r). The PSF modeling and image reconstruction results in a notable increase in the clarity and contrast of all resolved patterns. Moreover, some of the previously unresolved elements can consequently be distinguished (e.g. see Group −1 Element 6 image at 0.5 THz).

### C. BOEHLER STAR TEST TARGET

**FIGURE 6.**
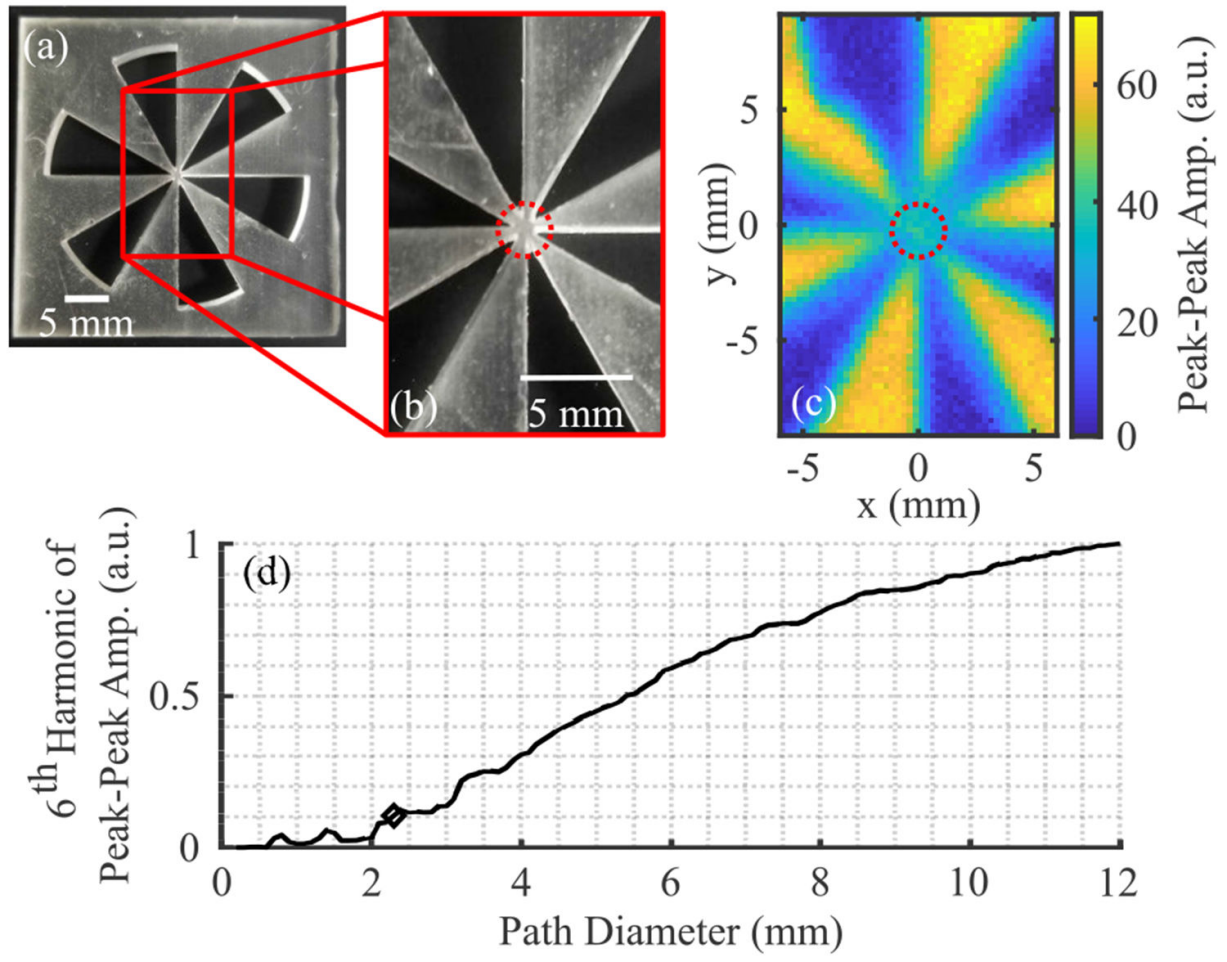
Boehler Star target and analysis. (a) Photograph of target. (b) Scanning area to scale with (c) image of peak-peak amplitude of reflected signal. (d) The sixth harmonic of amplitude along paths of different diameters, centered on the star pattern (normalized). The diamond marks the location where the plot crosses the 10% cutoff and correspond to the red circles in (b) and (c).

Additionally, to evaluate the performance of our THz PHASR scanner in different imaging scenarios we used a 6-petal Boehler Star-type target. The pattern, shown in Fig. 6(a-b), was 3D printed with 0.025 mm resolution in clear resin using a Formlabs Form 2 printer. The sample was nominally 3 mm thick. The size of the unresolved area at the center of the images provides a quantitative estimate of the resolution of the scanner [3], [38]. As opposed to the USAF target, the benefit of using the Boehler Star is that the resolution can be measured using a single pattern, however this method does not distinguish between the resolution in the horizontal and vertical directions. A peak-peak time-domain amplitude image is shown in Fig. 6(c). The Fourier transform of the signal amplitude along circular paths of different diameters, *d* (red dotted circle) are calculated. The 6th harmonic component of the Fourier transform (corresponding to the frequency of petals per circuit around the path) is plotted in Fig. 6(d). We define a threshold of 10% of the maximum value to determine the diameter at which the petals can be distinctly resolved. The approximate resolution is given by the arc length spanned by a petal at that diameter in the same way as the width of a single line does in the USAF target. That is, for an *N*-petal star the resolution is about *l* = *πd*/2*N*. Using this technique, the performance of the scanner in maintaining a consistent imaging resolution over the imaging plane was characterized.

**FIGURE 7.**
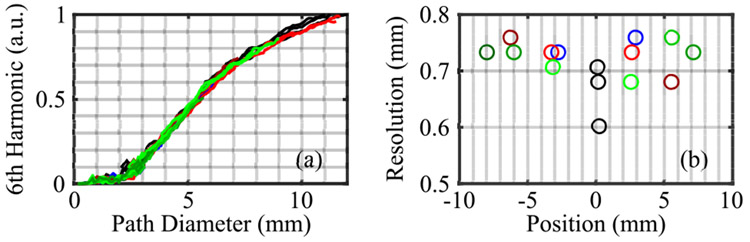
Boehler star measurements at different locations. (a) 6th harmonic of peak-peak amplitude, (b) the corresponding calculated resolutions as a function of Boehler Star position, where horizontal displacement is shown with blue, vertical with red, and diagonal with green markers. The three black markers show the variation in the resolution of a centered star image due to imaging noise.

To test the resolution consistency, the Boehler Star target was positioned at a variety of different locations over the FOV. THz images were obtained when the center of the Boehler Star was displaced by ±3 mm in the horizontal direction; ±3 and ±6 mm in the vertical direction; and ±3, ±6, and ±8 mm in the diagonal direction. The amplitude of the 6th harmonic was extracted from each image and used to calculate the resolution at each location, shown in Fig. 7(a). All traces have approximately the same trend, resulting in calculated resolutions between 0.60 mm and 0.76 mm, in agreement with the USAF target measurements. Fig. 7(b) shows results from three separate resolution measurements of a centered star with black markers, indicating a difference of approximately 0.1 mm due to the noise variation between these measurements. Fig. 7(b) also shows the spatial resolution for targets at different positions along the horizontal (blue markers), vertical (red markers) and diagonal (green markers) directions. The position is given by the location of the center of the star. The resolutions measured using displaced target images did not have any distinct dependence on the location, thereby confirming imaging resolution consistency over the entire FOV.

## IV. NON-DESTRUCTIVE TESTING APPLICATION

**FIGURE 8.**
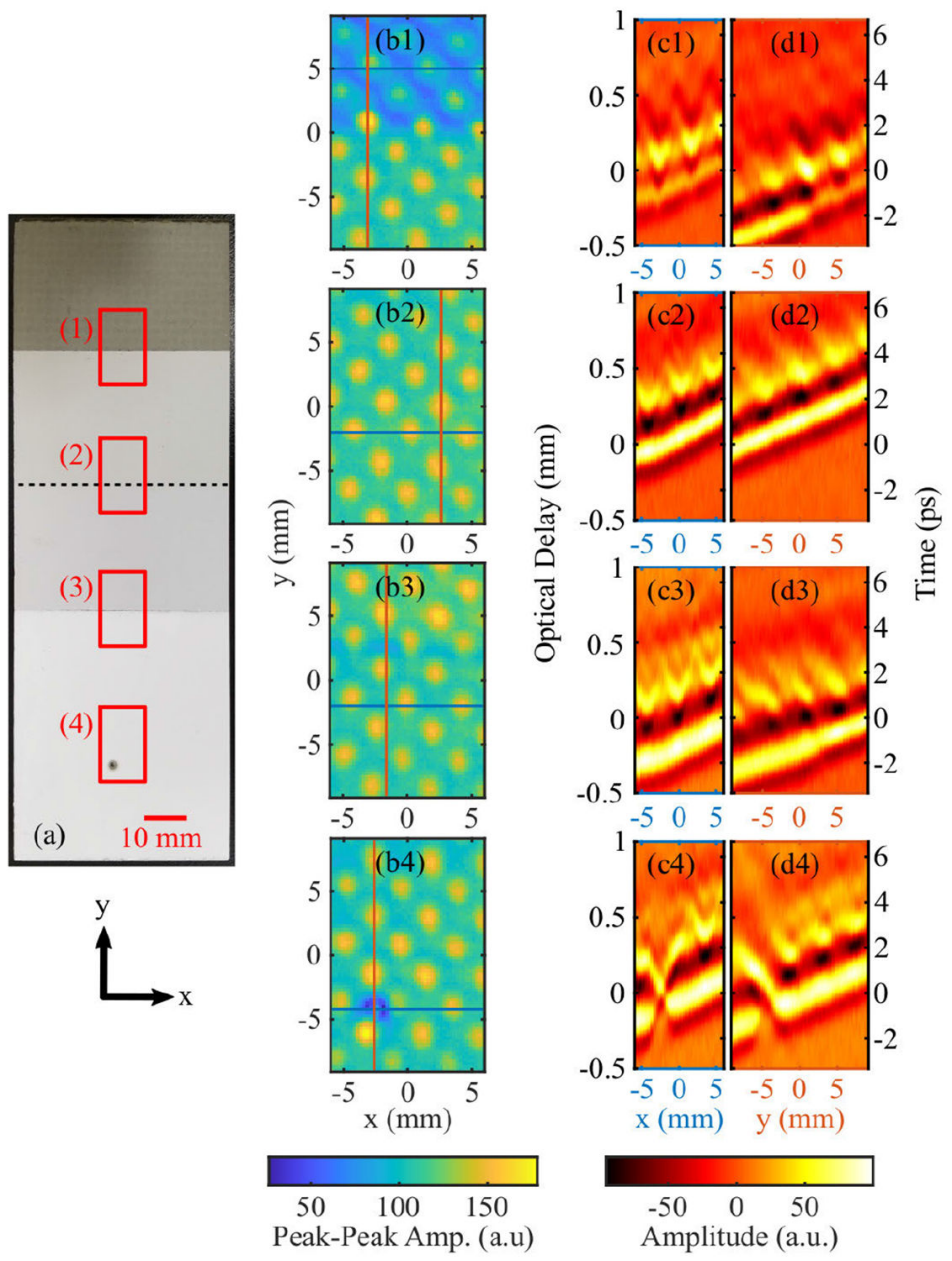
Airplane Wing Sample. (a) Photograph with red outlines showing the imaging areas and black dashed line to emphasize border between two coating sections. (b1-b4) Peak-Peak amplitude images corresponding to the red boxes 1-4 in part (a). (c) Horizontal and (d) vertical cross-sections showing the time-domain traces along the blue and red lines in (b1-b4), respectively.

Finally, we demonstrate the utility of our THz PHASR scanner in a non-destructive testing scenario by examining subsurface features of an airplane wing sample. This sample, shown in Fig. 8(a), is comprised of different protective coating layers covering an interwoven 6-layer carbon fiber substrate, which embeds a metallic mesh grid, designed for protecting the wing from static electricity and lightening. The peak-peak time-domain amplitude images shown in Fig. 8(b1-b4), were obtained from the corresponding red rectangles in Fig. 8(a). While the metallic mesh network was not visible underneath the opaque coatings in part (a), its lattice structure can be seen in Fig. 8(b1-b4). Also, box 4 features an intentional defect created during the fabrication of the test sample. Since the feature pattern is clearly resolved in Fig. 8(b), no additional signal processing was used for image enhancement. Cross-sectional time-domain traces along the horizontal (x, blue) and vertical (y, red) lines drawn in Fig. 8(b1-b4) are shown in Fig. 8(c1-c4) and (d1-d4), respectively. Parts (c) and (d) share the same vertical axis, where the left axis represents the optical delay (mm), whereas the right vertical axis represents time (ps). Notably, there is a clear change in the reflective properties in the transition line between different coatings in box 1 and 3, as shown by the cross-sectional time-domain traces in Fig. 8(d1 and d3). Additionally, a scan of the box 4 area clearly shows the location of the defect in both the peak-peak image, Fig. 8(b4), and the time-domain traces, Fig. 8(c4 and d4). It is evident that the defect extends into the substrate layer.

## V. CONCLUSION

The development of the THz PHASR scanner provides a portable solution for THZ-TDS imaging with approximately 1 THz bandwidth and about 100 waveforms/s recording speed. Our device has consistent resolution over the full 12 × 19 mm field of view. Additionally, we have demonstrated the application of a PSF modeling and deconvolution technique to enhance the achievable THz image clarity and contrast, and to improve spatial resolution. Finally, we demonstrated the application of the THz PHASR scanner in resolving sub-surface features in an aerospace sample. Portable and handheld THz-TDS scanners, such as the one presented in this article, can facilitate promising applications in non-destructive testing and biomedical imaging, where field-deployable technologies are needed.

## Supplementary Material

supp1-3045460

## Figures and Tables

**TABLE 1.** Contrast Values for Original Set of Images. Values Given Where Tribar Pattern was Properly Resolved

Resolution Direction	Group	Element	Feature Size (mm)	Frequency (THz)			
				0.3	0.5	0.7	1.0
Horizontal	−1	3	0.79	-	23%	48%	79%
		4	0.71	-	21%	39%	68%
		5	0.63	-	-	35%	75%
		6	0.56	-	-	25%	55%
	0	1	0.50	-	-	-	48%
Vertical	−1	3	0.79	-	-	23%	74%
		4	0.71	-	-	23%	61%
		5	0.63	-	-	-	50%
		6	0.56	-	-	-	47%
	0	1	0.50	-	-	-	-

**TABLE 2.** Contrast Values for Spatially Deconvolved Set of Images. Values Given Where Tribar Pattern was Properly Resolved

Resolution Direction	Group	Element	Feature Size (mm)	Frequency (THz)			
				0.3	0.5	0.7	1.0
Horizontal	−1	3	0.79	17%	61%	79%	84%
		4	0.71	-	56%	69%	87%
		5	0.63	-	37%	61%	95%
		6	0.56	-	16%	50%	81%
	0	1	0.50	-	-	48%	94%
Vertical	−1	3	0.79	-	-	51%	84%
		4	0.71	-	11%	49%	71%
		5	0.63	-	-	39%	54%
		6	0.56	-	-	-	59%
	0	1	0.50	-	-	-	-
