# Supplementary material for: Terahertz Portable Handheld Spectral Reflection (PHASR) Scanner
Source: IEEE Access. Author manuscript; Available in PMC 2022 Apr 14. (PMC9009755; doi:10.1109/access.2020.3045460)
Supplement: supp1-3045460 [file NIHMS1658433-supplement-supp1-3045460.pdf]

Supplemental Material: Terahertz Portable Handheld Spectral Reflection (PHASR) Scanner

1 Data Processing

Fig. 1 below shows a flowchart of the data processing steps we used for our PHASR Scanner images. The average of 20 raw terahertz time-domain measurements was recorded for each pixel. Post-processing was done in MATLAB. Initial denoising consisted of taking the discrete Fourier transform (using the fast Fourier transform, FFT, algorithm), applying a high-pass filter, and then taking the inverse FFT to return to the time-domain. This has the effect of removing the low-frequency background noise. Further denoising was accomplished using wavelet shrinkage with decomposition level-based thresholding as described in Ref. [38] of the main manuscript. A uniform set of manually defined windows were applied across all pixels within an image to isolate specific reflections. The maximum peak-peak amplitude within the window for each pixel provided a representative time-domain image.

To form initial frequency images, a split Blackman window was applied to the 25 points on the edge of each window and the FFT was calculated. The result, $i(x, y, f)$, is a series of amplitude images as measured at various frequencies. Images of the United States Air Force (USAF) 1951 negative resolution test target at select frequencies are presented in Fig. 4 of the main manuscript.

Fig. 5 of the main manuscript shows the same images of the USAF target after deconvolution by a modeled point spread function (PSF). The model is based on a reference time-domain measurement taken from a mirror located at the focus of the PHASR Scanner. Again, the average of 20 raw terahertz time-domain measurements was recorded and the same initial processing steps were applied. The time-domain window of the reference is the same length as the one applied to the image data. The result, $h(0, 0, f)$, provides the spectral content for the model. The spatial distribution of the PSF is then generated based on the lens characteristics using the technique presented in Ref. [42] of the main manuscript. The combination of these aspects provides the spot profile of each frequency, $h(x, y, f)$.

Wiener deconvolution of the frequency image by the PSF is done in the spatial frequency domain as described in the main manuscript and provides an estimate of the object function.

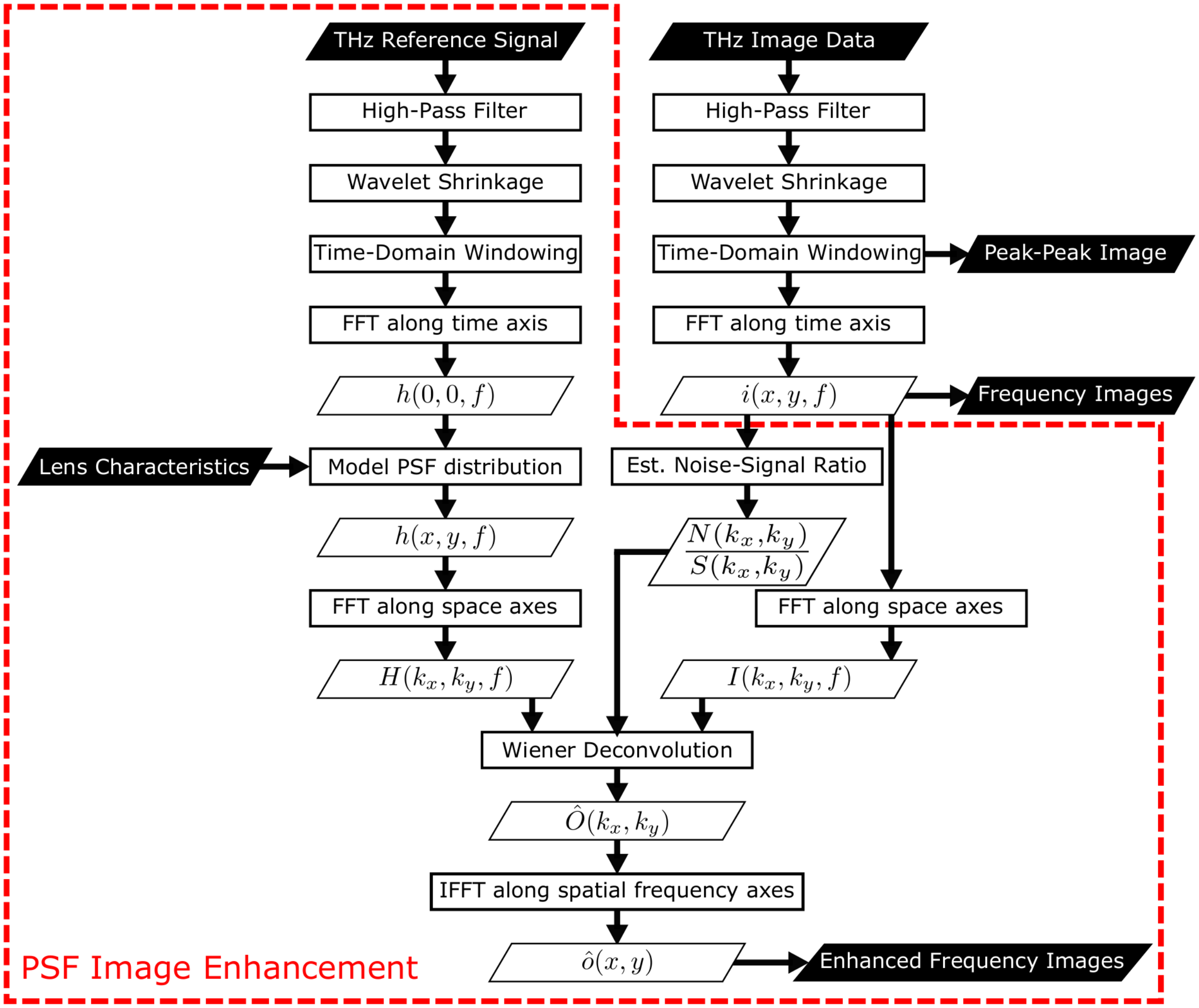

Figure 1: Flowchart showing data processing steps
